# Effect of Boiling in Water of Barley and Buckwheat Groats on the Antioxidant Properties and Dietary Fiber Composition

**DOI:** 10.1007/s11130-014-0425-x

**Published:** 2014-06-18

**Authors:** Marzanna Hęś, Krzysztof Dziedzic, Danuta Górecka, Agnieszka Drożdżyńska, Elżbieta Gujska

**Affiliations:** 1 Department of Food Service and Catering, Poznań University of Life Sciences, 60-637 Poznań, Poland; 2 Department of Biotechnology and Food Microbiology, Poznań University of Life Sciences, 60-627 Poznań, Poland; 3 Department of Food Science, University of Warmia and Mazury, 10-957 Olsztyn, Poland

**Keywords:** Barley groats, Buckwheat groats, Polyphenols, Antioxidant activity, Dietary fiber

## Abstract

In recent years, there has been an ever-increasing interest in the research of polyphenols obtained from dietary sources, and their antioxidative properties. The purpose of this study was to determine the effect of boiling buckwheat and barley groats on the antioxidant properties and dietary fiber composition. Antioxidative properties were investigated using methyl linoleate model system, by assessing the DPPH (2,2-diphenyl-1-picrylhydrazyl) radical scavenging activity and metal chelating activity. The results were compared with butylated hydroxytoluene (BHT). Raw barley and buckwheat groats extracts showed higher DPPH scavenging ability compared to boiled barley and buckwheat groats extracts. Raw barley groats extract exhibited higher antioxidant activity than boiled groats extract in the methyl linoleate emulsion. Higher chelating ability in relation to Fe (II) ions was observed for boiled groats extracts as compared to raw groats extracts. BHT showed small antiradical activity and metal chelating activity, while showing higher antioxidative activity in emulsion system. The analysis of groats extracts using HPLC method showed the presence of rutin, catechin, quercetin, gallic, *p*-hydroxybenzoic, *p*-coumaric, *o*-coumaric, vanillic, sinapic, and ferulic acids. Differences in the content of dietary fiber and its fractions were observed in the examined products. The highest total dietary fiber content was detected in boiled buckwheat groats, while the lowest - in boiled barley groats. The scientific achievements of this research could help consumers to choose those cereal products available on the market, such as barley and buckwheat groats, which are a rich source of antioxidative compounds and dietary fiber.

## Introduction

One of the possible modifications of dietary composition, aiming at improving its health promoting properties, is to increase the content of natural non-nutritive food substances with beneficial biological properties. In this group, apart from antioxidant vitamins, carotenoids, minerals and dietary fiber, we can classify low-molecular-weight secondary metabolites of plants [1, 2].

Cereals and pseudo cereals are an important source of macronutrients and bioactive substances with antioxidant activity [3]. Growing interest in barley has been observed recently, due to its high levels of soluble fiber and phenolic compounds such as: benzoic and cinnamic acid derivatives, proanthocyanidins, quinines, flavonols, chalcones, flavones, flavanones, and amino phenolic compounds. The major phenolic acid found in barley is ferulic acid [4, 5]. Therefore, barley can be an excellent source of natural antioxidants to inhibit lipid oxidation, or for disease prevention and health promotion [6]. Buckwheat grain contains proteins with high biological value and balanced amino acid composition, relatively high dietary fiber content and vitamins B1, B2, B6, rutin and quercetin, the content of which changes depending on technological parameters applied in seed processing [1, 7].

The technological production process of buckwheat and barley groats includes such stages as cleaning and thermal conditioning (roasting) of grains, size sorting, dehulling, sorting after dehulling and sorting of groats connected with the separation of waste and by-products [8]. The most common form of processing groats is boiling in water. The boiled final product can have various levels of consistency – porridge-like, crumbly or loose. The most beneficial method of preparation, which preserves all nutritive values, is boiling groats loose, so that water is completely absorbed. There are very few studies describing antioxidative action of polyphenols derived from buckwheat or barley seeds and buckwheat or barley products, and determining the effect of thermal processing on their activity. Thus, the aim of this work was to determine the effect of boiling on dietary fiber composition and antioxidant activity of phenolic compounds in barley and buckwheat groats.

## Materials and Methods

### Materials

Grains of barley Antek cultivar were obtained from Breading Station Danko (Poland) and were used for production of barley groats. Buckwheat grains (*Fagopyrum esculentum* Moench), Kora cultivar were obtain from Breading Station Palikije (Poland) and were used for production of buckwheat groats. Raw buckwheat groats were roasted and dehulled in industrial environment. Barley and buckwheat groats were used both as raw as well as boiled material. The groats were boiled with water/groats ratio of 2:1 (v/v), for 30 min until water has been completely absorbed. After boiling the groats were lyophilized and then comminuted in a Cyclotec mill.

### Chemicals

The following chemicals were used: 2,2-diphenyl-1-picrylhydrazyl (DPPH), Folin-Ciocalteu reagent (FCR), (+) catechin, 3-(2-pyridyl)-5,6-bis (4-phenyl-sulfonic acid)-1,2,4-triazine (Ferrozine), Tween 20, α-amylase, pepsin, pancreatin, phenolic acids: *o*-coumaric, *p*-coumaric, ferulic, sinapic, vanillic, gallic, and *p*-hydroxybenzoic; flavonoids: catechine, quercetin, and rutin were obtained from Sigma-Aldrich (Germany); acetone, methanol, ethanol, sodium carbonate POCH (Poland), methyl linoleate Nu-Chek Prep. (USA); BHT Merck (Germany), thermostable α-amylase from Novozymes (Denmark). All other chemicals were of analytical grade.

### Chemical Analysis

Phenolic compounds were extracted from ground samples according to Amarowicz *et al.* [9] with 80 % (v/v) aqueous acetone at 80 °C for 15 min at a solid to solvent ratio of 1:10 (w/v). The content of total phenolic compounds in extract was estimated using the Folin-Ciocalteu reagent (FCR) [10]. Aliquot of extract (0.5 mL) was added to 8 mL distilled water and 5 mL FCR. The mixture was mixed with 1 mL of saturated sodium carbonate solution. After incubation at room temperature for 60 min, the absorbance of the mixture was read at 750 nm. Results are expressed as mg of (+) catechin equivalents per gram of dry matter extract (mg CE/g).

The content of flavonoids and phenolic acids was estimated using the method described by Drożdżyńska *et al.* [11]. Fast liquid chromatography (FLC) was performed with an Agilent Technologies 1200 series system. Chromatograms were recorded at 280 nm for gallic acid, vanillic acid, *p*-hydroxybenzoic acid, and catechin at 320 nm for *p*-coumaric, *o*-coumaric, sinapic, ferulic acids, and at 360 nm for rutin and quercetin.

The ability to inhibit autoxidation of methyl linoleate was determined following Lingnert *et al.* [12]. The method consisted of the spectrophotometric (λ = 234 nm) determination of the increment in the contents of conjugated dienes in methyl linoleate emulsion after 19 h of incubation in the dark at 37 °C. Antioxidant efficiency coefficient (AEC) was expressed as the ratio of the increment in absorbance of the control sample and tested sample to the increase in absorbance of the control sample.

The capacity of the prepared extract to scavenge the stable free radical 2,2-diphenyl-1-picrylhydrazyl (DPPH) was monitored according to the method of Sanchez-Moreno *et al.* [13]. DPPH (1 mM, 0.25 mL) was dissolved in pure methanol and was added to 0.1 mL of polyphenol extracts with 2 mL of methanol. The decrease in absorbance of the resulting solution was determined at 517 nm at 30 min.

The chelation of ferrous ions by extracts was estimated using the method of Tang *et al.* [14]. The assay consisted of the colorimetric measurements of the degree of discolouration of iron (II) chloride (2 mM, 0.1 mL) complexes with ferrozine (5 mM, 0.2 mL) caused by the extracts. The applied wavelength was 562 nm.

The content of total dietary fiber (TDF), soluble dietary fiber (SDF) and insoluble dietary fiber (IDF) was estimated according to Asp *et al.* [15]. Dietary fiber was determined under conditions similar to those found in the human alimentary tract using the following enzymes: thermostable α-amylase (pH 6.0, 90 °C, Termamyl 120 L), pepsin (pH 1.5, 40 °C) and pancreatin (pH 6.8, 40 °C). The contents of neutral dietary fiber (NDF), acid detergent fiber (ADF), lignin and cellulose were determined using the detergent method according to Van Soest [16], as modified by McQueen and Nicholson [17].

### Statistical Analysis

All data were subjected to analysis of variance (ANOVA) using the general linear models of statistical analysis system software (version 8). The significant differences among multiple groups were determined by Tukey’s test (*P* < 0.05). Spearman’s rank correlation was used to determine the correlation coefficient between the content of phenolic compounds and the content of fiber and its fractions in groats, and their antioxidative properties.

## Results and Discussion

The total phenolic content in buckwheat groats was higher than in the barley groats (Table 1). Boiling affected the total content of phenolic compounds in groats. Extracts of boiled buckwheat groats showed a significantly higher content of polyphenols than raw buckwheat groats. However, extracts of raw barley groats contained more total phenolic in comparison to boiled groats. Statistical analysis showed a correlation of polyphenols in groats with antioxidant activities (*r* = 0.80 for DPPH scavenging activity, antioxidant efficiency coefficient and chelating activity). Boiling of barley and buckwheat groats also affected the content of flavonoids and phenolic acids (Table 2). Boiled buckwheat groats contained significantly more catechins in comparison to raw buckwheat groats, respectively 62.12 and 46.47 mg/kg DM. extract and significantly less *p*-coumaric acid (0.91 mg/kg DM extract) in comparison to raw buckwheat (7.26 mg/kg DM extract). Heat treatment of barley groats caused a decrease in the content of sinapic acid (19 %) in comparison to raw barley groats and a slight increase in ferulic acid content (3 %). Boiling of buckwheat groats did not induce any change in the content of rutin. Higher content of polyphenols in boiled buckwheat groats was possibly due to their partial release from the bound form with proteins as a result of boiling. Moreover, phenolics may also be associated with other plant components such as carbohydrates. Technological processing can modify the polyphenol content of foods in several ways [18]. The influence of processing on the content of phenolic compounds and antioxidant activity of vegetables, fruits, seeds of legume plants and cereals is not unequivocal, and changes in antioxidant activity of the technological processes depend on the raw material used and the processing conditions, mainly the time and temperature of the process [18]. Kreft *et al.* [19] observed a decreased content of rutin after boiling buckwheat groats for 60 min. In research studies the manually hulled seeds were used as material. Studies conducted by Zhang *et al.* [20] showed that roasting, pressured-steam heating and microwave heating of buckwheat flour caused a decrease in total phenolics, total flavonoids and antioxidative activities. A significant decrease in the total phenolic content and total flavonoid content was observed upon extrusion [21]. On the other hand, it has been demonstrated that the process of boiling can lead to increased phenolic content in green beans, bell pepper and broccolis [22] and an increased level of free flavonols in tomatoes [23]. Zieliński *et al.* [24] observed a five-fold increase in the content of ferulic acid in cereal material upon extrusion.

**Table 1:** Total phenolic content in groats extracts

Sample	Total phenolic (mg CE^*^/kg DM extract)
Raw barley groats	6800 ± 50^b^
Boiled barley groats	6100 ± 70^a^
Raw buckwheat groats	9200 ± 100^c^
Boiled buckwheat groats	13500 ± 120^d^

* CE - catechin equivalents; different letters indicate significant differences (*P < 0.05*). Mean values ± SD (*n* = 3)

**Table 2:** Polyphenol content in groats extracts

Phenolics substances (mg/kg DM extract)	Raw buckwheat groats	Boiled buckwheat groats	Raw barley groats	Boiled barley groats
Rutin	69.95 ± 2.25^a^	70.48 ± 3.27^a^	nd	nd
Catechin	46.47 ± 0.17^a^	62.12 ± 6.04^b^	1312.47 ± 7.11^c^	1321.58 ± 6.20^c^
Quercetin	1.67 ± 0.03^a^	1.48 ± 0.16^a^	nd	nd
*p*-Coumaric acid	7.26 ± 0.11^b^	0.91 ± 0.02^a^	42.16 ± 1.10^c^	49.61 ± 1.75^d^
*o*-Coumaric acid	nd	nd	28.29 ± 1.25^a^	29.94 ± 1.15^a^
Vanillic acid	nd	nd	4.77 ± 0.11^a^	4.76 ± 0.09^a^
Sinapic acid	nd	nd	19.23 ± 1.53^b^	15.62 ± 1.25^a^
Ferulic acid	nd	nd	84.92 ± 2.30^a^	87.37 ± 2.15^a^
*p*-Hydroxybenzoic acid	22.06 ± 1.55	nd	nd	nd
Gallic acid	3.83 ± 0.08^a^	4.08 ± 0.10^a^	nd	nd

*nd* – not detected; different letters in the same row indicate significant differences (*P < 0.05*). Mean values ± SD (*n* = 3)

**Fig. 1:**
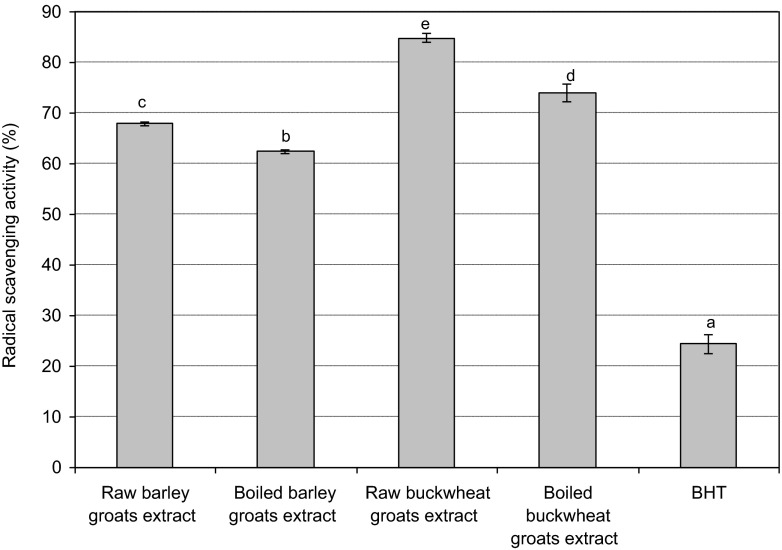
The DPPH scavenging of groats extracts. Different letters indicate significant differences (*P < 0.05*). Mean values ± SD (*n* = 4)

The free radical 2,2-diphenyl-1-picrylhydrazyl (DPPH) scavenging activity for different kinds of groats extract are shown in Fig. 1. Higher DPPH radical scavenging capacity was found for the extracts of raw buckwheat (84.8 %) and barley (67.9 %) groats in comparison to boiled buckwheat and barley groats, 74.1 and 62.4 %, respectively. Significantly weaker antiradical properties (24.4 %) were found for butylated hydroxytoluene (BHT). The antioxidative compounds in food can interact, which leads to unforeseeable changes in the levels of antioxidative activity. It has also been put forward that, in addition to phenolic compounds, other buckwheat components influence its activity. This could explain a lower free radical DPPH scavenging activity of boiled buckwheat groats, where a higher polyphenol content was assayed. Food processing can further influence these changes in activity levels [25, 26].

**Fig. 2:**
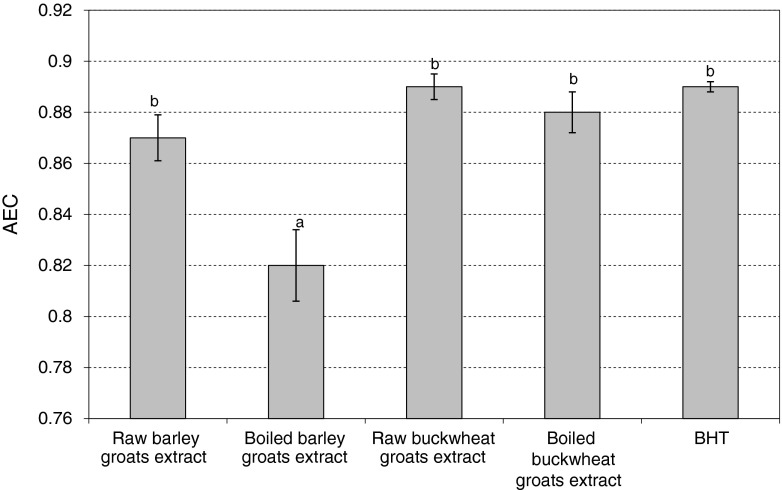
Antioxidant activity of groats extracts in methyl linoleate emulsion. *AEC* – Antioxidant efficiency coefficient. Different letters indicate significant differences (*P < 0.05*). Mean values ± SD (*n* = 4)

Figure 2 shows the effect of extract on the stability of methyl linoleate in emulsion. Antioxidant efficiency coefficient (AEC) showed high antioxidant activity of groats extracts. Significantly higher ability to inhibit autoxidation of methyl linoleate was exhibited by the extract of raw barley groats (AEC = 0.87) than its boiled form (AEC = 0.82). Both extracts of buckwheat groats very actively inhibited oxidative changes in the emulsion. Their activity was comparable to BHT (AEC = 0.89). Sun and Ho [27] found that the methanolic extract of buckwheat seed inhibited oxidation of β-carotene emulsion more effectively than the acetone, butanol or ethanol extracts; however, the activity of the analyzed extracts was much lower than that of BHT and BHA (butylated hydroxyanisole).

**Fig. 3:**
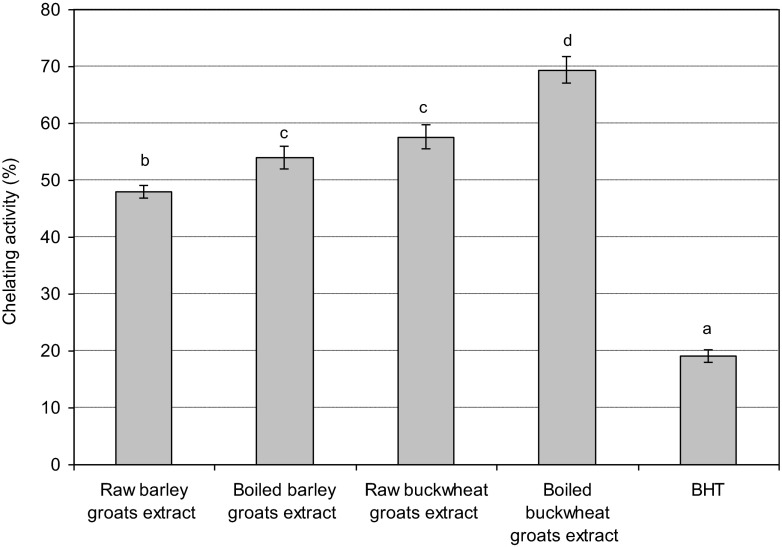
The chelating activity of groats extracts. Different letters indicate significant differences (*P < 0.05*). Mean values ± SD (*n* = 4)

The ability of the extracts to chelate iron ions was presented in Fig. 3. The type of extract significantly affected the capacity to form iron ion complexes. Among groats extracts a higher capacity of Fe(II) ion chelating was found for extracts of boiled groats (barley groats – 53.9 %, buckwheat groats – 69.4 %) than raw groats, 48.0 and 57.6 %, respectively. A slight activity was shown by BHT (19.1 %). The metal chelating capacity may significantly influence the course of oxidative reactions, thus metal binding compounds are included in the class of oxidation inhibitors.

The antioxidant activity of extracts is not always dependent on the level of phenolic compounds, but can be different depending on used methods [28]. Therefore it is difficult to compare results obtained using different methods; however, they indicate a diversity of mechanisms of action of antioxidants. A decreased content of natural antioxidants in a product can be accompanied by their higher antioxidative activity, due to more easily accessible antioxidants of other types [26]. According to Grajek [29], the beneficial influence of short-lived thermal processing on the antioxidative activity can be attributed to the removal of oxygen, denaturation of enzymes from the oxidoreductase group and the transition of the antioxidant into a more active form (aglycone). Studies conducted by Sensoy *et al.* [18] showed that roasting at 200 °C for 10 min slightly decreased in buckwheat flour the radical scavenging activity, whereas extrusion (170 °C) did not cause any change. The results suggest that processing conditions can be optimized to retain the health promoting compounds in products. The reason for reducing antioxidant activity in processing of raw materials could include oxidation of antioxidant, complexation with other food ingredients, enzymatic modification, increased oxidative capacity of the environment and the transition from antioxidant to pro-oxidant. Oxidation processes are particularly dangerous [25, 29]. The boiling of plant material could lead to rapid heat transfer into the tissue and enlarge losses of antioxidants, and to partial extraction of hydrophilic antioxidants into water, decreasing the antioxidant capacity of boiled samples [30].

The highest content of total dietary fiber (TDF) was shown in boiled buckwheat groats (16.45 %), while the lowest in boiled barley groats (7.99 %) (Table 3). The highest level of insoluble dietary fiber (IDF) was found in boiled buckwheat groats (13.10 %), while the lowest in boiled barley groats (5.19 %) (Table 3). The content of the soluble dietary fiber (SDF) fraction ranged from 2.21 % (raw buckwheat groats) to 4.56 % (raw barley groats).

**Table 3:** Content of TDF, NDF and its fractions

Fraction of dietary fiber (g/100 g product)	Raw buckwheat groats	Boiled buckwheat groats	Raw barley groats	Boiled barley groats
Total dietary fiber				
TDF	12.18 ± 0.24^b^	16.45 ± 0.26^d^	13.21 ± 0.39^c^	7.99 ± 0.36^a^
IDF	9.97 ± 0.22^c^	13.10 ± 0.16^d^	8.65 ± 0.34^b^	5.19 ± 0.17^a^
SDF	2.21 ± 0.02^a^	3.35 ± 0.22^c^	4.56 ± 0.09^d^	2.80 ± 0.14^b^
Neutral dietary fiber				
NDF	10.00 ± 0.72^c^	11.26 ± 0.47^c^	7.06 ± 0.07^b^	4.22 ± 0.40^a^
C	0.67 ± 0.12^a^	2.34 ± 0.54^b^	4.00 ± 0.26^c^	0.78 ± 0.37^a^
H	6.94 ± 0.61^b^	2.69 ± 0.61^a^	2.84 ± 0.33^a^	3.31 ± 0.07^a^
L	2.39 ± 0.01^b^	6.24 ± 0.23^c^	0.22 ± 0.14^a^	0.12 ± 0.02^a^

*TDF* – total dietary fiber; *IDF* - insoluble dietary fiber; *SDF* - soluble dietary fiber; *NDF*- neutral detergent fiber; *C* - cellulose; *H* - hemicellulose; *L* – lignin; different letters in the same row indicate significant differences *(P < 0.05)*. Mean values ± SD (*n* = 3)

The groats showed varied content of dietary fiber fractions. The highest lignin level was found in boiled buckwheat groats (6.24 %), while the lowest in boiled barley groats (0.12 %). The highest content of cellulose was observed for raw barley groats (4.00 %), while the lowest for raw buckwheat groats and boiled barley groats, 0.67 and 0.78 %, respectively. A significantly higher content of hemicelluloses was found in raw buckwheat groats (6.94 %) in comparison with other groats. It was shown that the content of IDF, neutral detergent fiber (NDF) and lignin in groats was correlated with antioxidant activities (*r* = 0.80).

A study by Gualberto *et al.* [31] indicates that changes in the content of SDF and IDF depend on the type of the cereal raw material. Those authors showed no effect of extrusion on the content of dietary fiber of wheat bran, while in case of oat and rice bran they found a slight decrease in the content of the IDF fraction and an increase in the content of the SDF fraction. An increase in the content of dietary fiber, mainly the lignin fraction, may be related to Maillard’s reaction products being formed during thermal processing, which in the Van Soest method are determined as the lignin fraction [32]. A similar trend was also observed by Valiente *et al.* [33], and Dziedzic *et al.* [8].

## Conclusions

Barley and buckwheat groats are products very rich in bioactive substances such as dietary fiber and polyphenols. Boiling for 30 min in 2:1 (water:groats) ratio does not affect the nutritional properties of buckwheat groats in any negative way. It should be emphasized that this is the recommended way of cooking buckwheat groats. Extracts of boiled buckwheat groats showed a significantly higher content of polyphenols and total dietary fiber than raw buckwheat groats. Both boiled groats extracts formed complexes with iron ions to a higher degree than raw groats extracts. Buckwheat and barley have a high potential as materials for functional food development and production. They would be an ideal possible base for the design of novel foods.
